# The use of large language models in generating multiple choice questions for health professions education: A systematic review and network meta-analysis

**DOI:** 10.1371/journal.pone.0340277

**Published:** 2026-01-02

**Authors:** Lauren Riehm, Keean Nanji, Moiz Lakhani, Evelina Pankiv, Dean Hasanee, Wesla Pfeifer

**Affiliations:** 1 Department of Anesthesia and Pain Medicine, The Hospital for Sick Children, Toronto, Ontario, Canada; 2 Department of Anesthesiology and Pain Medicine, University of Toronto, Toronto, Ontario, Canada; 3 Division of Ophthalmology, Department of Surgery, McMaster University, Hamilton, Ontario, Canada; 4 Department of Health Research Methods, Evidence and Impact, McMaster University, Hamilton, Ontario, Canada; 5 Faculty of Medicine, University of Ottawa, Ottawa, Ontario, Canada; 6 Department of Surgery, McMaster University, Hamilton, Ontario, Canada; The University of Texas Rio Grande Valley School of Medicine, UNITED STATES OF AMERICA

## Abstract

**Purpose:**

Large language models (LLMs) have the potential to change medical education. Whether LLMs can generate multiple-choice questions (MCQs) that are of similar quality to those created by humans is unclear. This investigation assessed the quality of MCQs generated by LLMs compared to humans.

**Methods:**

This review was registered with PROSPERO (CRD42025608775). A systematic review and frequentist random-effects network meta-analysis (NMA) or pairwise meta-analysis was performed. Ovid MEDLINE, Ovid EMBASE, and Scopus were searched from inception to November 1, 2024. The quality of MCQs was assessed with seven pre-defined outcomes: question relevance, clarity, accuracy/correctness; distractor quality; item difficulty analysis; and item discrimination analysis (point biserial correlation and item discrimination index). Continuous data were transformed to a 10-point scale to facilitate statistical analysis and reported as mean differences (MD). The MERSQI and the Grade of Recommendations, Assessment, Development and Evaluation (GRADE) NMA guidelines were used to assess risk of bias and certainty of evidence assessments.

**Results:**

Five LLMs were included. NMA demonstrated that ChatGPT 4 generated similar quality MCQs to humans with regards to question relevance (MD −0.13; 95% CI: −0.44,0.18; GRADE: VERY LOW), question clarity (MD −0.03; 95% CI: −0.15,0.10; GRADE: VERY LOW), and distractor quality (MD −0.10; 95% CI: −0.24,0.04; GRADE: VERY LOW); however, MCQs generated by Llama 2 performed worse than humans with regards to question clarity (MD −1.21; 95% CI: −1.60,-0.82; GRADE: VERY LOW) and distractor quality (MD −1.50; 95% CI: −2.03,-0.97; GRADE: VERY LOW). Exploratory post-hoc t-tests demonstrated that ChatGPT 3.5 performed worse than Llama 2 and ChatGPT 4 with regards to question clarity and distractor quality (p < 0.001).

**Conclusion:**

ChatGPT 4 may create similar quality MCQs to humans, whereas ChatGPT 3.5 and Llama 2 may be of worse quality. Further studies that directly compare these LLMs to human-generated questions and administer MCQs to students are required.

## Introduction

### Rationale

Large Language Models (LLMs) are language-based foundation models that can comprehend and generate natural language, as well as participate in human-like conversations [1]. There are a variety LLMs which can be utilized for diverse purposes; since the release of ChatGPT in November 2022, there are many published studies evaluating the ability of LLMs to perform on medical licensing exams, such as the USMLE [2,3] and various subspecialty examinations (including neurology, neurosurgery, and ophthalmology board exams) [4–6]. Recently, there has been growing interest for the use of LLMs in the field of medical education [1,7].

LLMs can simplify complex topics from academic materials and provide feedback with scientifically backed explanations, making them an invaluable tool to prepare revision aids and test the foundational knowledge of medical learners [2,3,8]. LLMs are invaluable for medical educators as they enhance the ability to rapidly obtain and assemble information; this reduces the time required to develop educational resources and improves overall efficiency in environments where medical educators must balance their limited time between education and clinical duties [9]. LLMs may be useful in creating and reviewing multiple choice questions (MCQs), as they are able to enhance comprehension for medical learners by providing explanations and reasoning for incorrect options [10]. In addition to their role in acquiring knowledge, there has been growing interest in the role of LLMs in generating examination material and testing learners’ knowledge acquisition. The MCQ has classically been a popular tool to test factual knowledge in a variety of fields; more recent guidelines within the field of medical education have emphasized ways the MCQ can be developed to test higher-order concepts, including knowledge integration, interpretation, and problem-solving [11].

It is unclear whether MCQs generated by LLMs are of comparable quality to those generated by humans; for example, whether they are of an appropriate level of difficulty, clearly written, able to discriminate between low- and high-performing students, or factually accurate. LLMs are plagued with issues such as hallucination, where the LLM produces content that diverges from the source material input by the user (input-conflicting hallucination), is in direct contradiction with information previously produced by itself (context-conflicting hallucination) or is not consistent with factual knowledge (fact-conflicting hallucination) [12]. It is integral that MCQs generated by LLMs are compared to those written by humans to assess whether the exam questions generated by LLMs are appropriate to use in medical examinations. However, there are few published studies evaluating the quality of MCQs written by LLMs, and even fewer comparing the quality of LLM-generated questions to those written by humans. A direct comparison of available LLM systems to determine which is most reliable has not yet been conducted. Previous reviews on this topic did not conduct any network meta-analysis and had search strategies that are now outdated [13].

### Objectives

The focus of this systematic review will be to address the following question: in health professions education, what is the quality of multiple-choice questions generated by the most commonly used large language models compared to those generated by humans? Specifically, which LLMs produce MCQs of comparable quality to human-written items? Quality will be evaluated based on the following outcomes: question relevance, question clarity, distractor quality, question accuracy/correctness, item difficulty analysis, and item discrimination analysis (point biserial correlation and item discrimination index).

## Methods

This systematic review was reported according to the Preferred Reporting Items for Systematic Reviews and Meta-Analysis (PRISMA) statement [14]. (S1 Table) and was registered with PROSPERO (CRD42025608775). Ethics approval was not required for this systematic review, as all analyses were performed using study data that had previously obtained informed consent and ethics approval.

### Eligibility criteria

Inclusion and exclusion criteria were created utilizing the PICOTS framework [15]. Studies were included if they involved the generation of MCQs by LLMs or humans for undergraduate and postgraduate learners from allied health care professions (including medicine, dentistry, pharmacy, nursing, occupational therapy, physiotherapy, and optometry). Studies were excluded if they involved the generation of questions for non-medical learners or if the exam questions were in a non-MCQ format. All data from published RCTs, cohort studies, case control studies, case series, commentaries/letters to the editor, and conference proceedings were included. Review articles were excluded.

### Information sources and search strategy

A literature search of Ovid MEDLINE, Ovid EMBASE, and Scopus databases was performed on November 1, 2024, for studies in all allied health care professions meeting our eligibility criteria. This search strategy was jointly developed by a multidisciplinary team comprised of clinicians and health research librarians. To identify any potentially overlooked studies, the reference list of screened studies was searched for further eligible studies. The as “Similar Articles” sections was also searched in their respective database for additional studies. Covidence (Veritas Health Innovation, Melbourne, Australia) was utilized to house exported studies for further screening. S1 File [16–18]. outlines the search strategy utilized for each database and registry.

### Selection process

Two reviewers (LR and ML) worked independently and in duplicate to screen the titles and abstracts, and subsequently all full texts, of studies that were identified during the initial search. Any discrepancies were discussed amongst reviewers to reach unanimous agreement. Covidence (Veritas Health Innovation, Melbourne, Australia) was used to track triage decisions.

### Data collection

Three reviewers (LR, ML, and DH) underwent a calibration and subsequently worked independently and in duplicate to extract data utilizing a standardized pilot-tested collection form designed in Microsoft Excel (Version 16.92). Discrepancies in data collection were resolved through a third party (KN). Any unclear information from the published manuscripts was clarified with the corresponding author.

### Data items

Information extracted regarding study design included the following: author(s), year of publication, journal of publication, the medical field MCQs tested upon (including subspecialty), the LLM(s) utilized including version, the presence of control MCQs generated by humans, the number of MCQs generated by the LLM(s) and humans, whether LLM quality was evaluated by humans and the qualifications of the individuals performing the evaluation, whether the MCQs were administered to students, the outcomes of interest, and risk of bias items.

### Outcome measures

To determine the quality of MCQs generated by LLMs, a literature review was performed [19,20] and seven pre-defined outcomes were identified: question relevance, question clarity, distractor quality, question accuracy/correctness, item difficulty analysis, and item discrimination analysis (point biserial correlation and item discrimination index). The item difficulty analysis assesses the proportion of examinees who respond correctly to a given item, whereas the item discrimination index reflects how well a given item distinguishes between high- and low-scoring students.

### Risk of bias

Two reviewers (LR and ML) underwent calibration, training and subsequently worked independently and in duplicate to assess the methodological quality of included studies using the MERSQI [21]. Discrepancies were resolved through a third party (KN). The quality of each medical education study was assessed across the following domains: study design, sampling (number of institutions and response rate), type of data (subjective or objective), validity (internal structure, content, and relationships to other variables), data analysis (appropriateness and complexity), and outcomes. Studies with a MERSQI score less than 11.6, as per Cook et al, were classified as low methodologic quality [22].

### Statistical analysis

The method of evidence synthesis for each outcome depended on the availability of data. In the event of sufficient data, a frequentist network meta-analysis using random effects modelling was performed comparing the results across the different LLMs and human control. The network meta-analysis allowed comparison of multiple different LLMs, even if particular LLMs were not directly compared in any study. Frequentist NMAs assume specified heterogeneity whereas Bayesian approaches incorporate a degree of uncertainty in the estimation of heterogeneity. Despite these differences, both are valid approaches as a study that re-analyzed NMAs using both methods found no major differences in the results obtained [23]. A frequentist approach was chosen in this analysis given that our group had more experience with this framework. If there was insufficient data for a network meta-analysis, random effects pairwise meta-analyses were performed comparing the LLMs to a human control. If all the available data were not incorporated into the network meta-analysis or pairwise meta-analysis, then random-effect single-arm analyses were performed to calculate pooled summary effect estimates across all LLMs or human controls.

Data reported on a Likert scale was transformed to a 10-point scale to facilitate statistical analyses. For continuous outcomes, the summary effect estimates reported the pooled mean difference with corresponding 95% confidence interval (CI) for comparative analyses and the pooled mean for single arm analyses. For dichotomous outcomes, relative risks were calculated and reported with their corresponding 95% CI. For outcomes reporting correlation coefficients, data were transformed to Fisher’s z scale to stabilize the variance and normalize the distribution prior to meta-analysis [24].

Complete case analyses were performed for all analyses. In the event of missing measures of variability for continuous outcome measures, standard deviations were imputed in line with the methodology from the Cochrane Handbook of Systematic Reviews [25].

Analyses were performed in R 4.0.4 (The R Project, Aukland, New Zealand) using the “meta”, and “netmeta” packages.

### Confidence in cumulative evidence

The certainty of the evidence was assessed following the Grade of Recommendations, Assessment, Development and Evaluation (GRADE) guidelines [26]. The GRADE approach involves separate grading of the quality of evidence for each outcome followed by determining an overall quality of evidence across outcomes. Two reviewers rated each domain for each comparison separately and resolved discrepancies by consensus. The certainty for each comparison and outcome was rated as ‘high’, ‘moderate’, ‘low’ or ‘very low’, based on the type of studies included, risk of bias, inconsistency, indirectness, publication bias, intransitivity, incoherence, and imprecision. Node splitting models were used to assess for incoherence [27]. Heterogeneity was assessed through a combination of visually inspecting the forest plot and assessing the I^2^ statistic. Publication bias was assessed by visually inspecting funnel plots and Egger’s test for comparisons informed by more than 10 studies. If there were fewer than 10 studies, publication bias was assessed by evaluating the quality of the search and measures taken to obtain all possible evidence to inform the outcome of interest.

### Subgroup analyses

Subgroup analyses were pre-specified in the protocol registration and were performed for all single-arm analyses to compare the effects of the different LLMs. If the chi-squared test for the subgroup analysis was significant, exploratory post-hoc t-tests were conducted comparing results between question sources. A correction for multiple hypothesis testing was not performed given that the post-hoc tests were strictly exploratory in nature.

## Results

### Study selection

**Fig 1:**
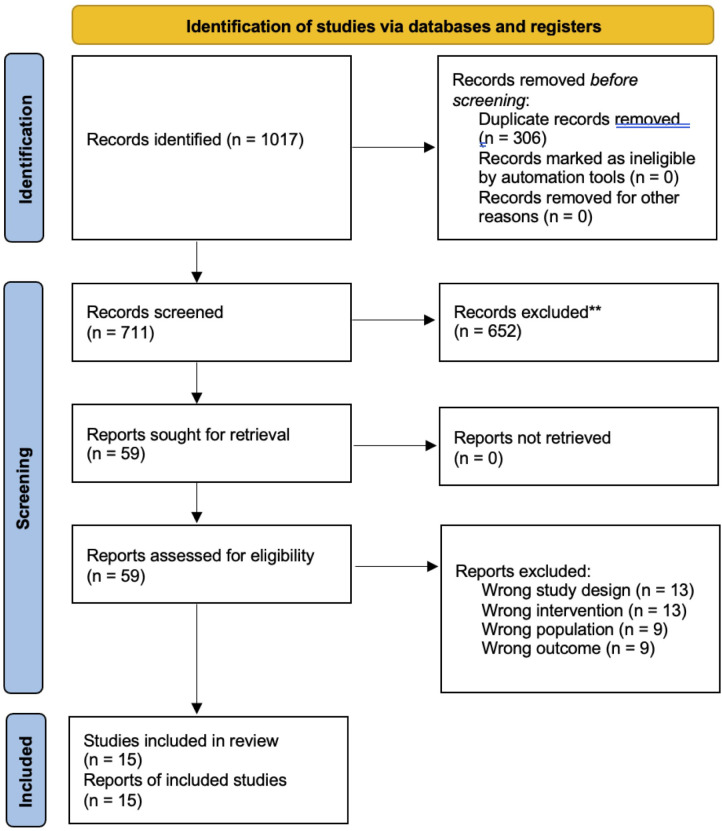
PRISMA flow diagram.

The Preferred Reporting Items for Systematic Reviews and Meta-Analysis flow diagram for study analysis is outlined in Fig 1. A total of 1,017 articles were identified through the database search; 306 were removed as duplicates, leaving 711 remaining articles that proceeded to title and abstract screening. Fifty-nine articles underwent full-text screening. Ultimately, 15 [28–42] studies met the review’s inclusion criteria.

### Study characteristics

Table 1 outlines the key characteristics of the included studies. The most frequently employed LLM was ChatGPT, with 10 studies utilizing ChatGPT 3.5 and 4 studies utilizing ChatGPT 4. Other LLMs used to create MCQs included Bard 1.0, Llama 2, and ChatPDF.com. Two studies compared the quality of MCQs generated by different LLMs, one of which additionally compared the quality of MCQs generated by different LLMs to those created by humans. Only 5 (33%) of the included studies compared MCQs created by LLMs to those created by humans. Eight (53%) studies administered the generated MCQs to students. Thirteen (87%) of the studies used a panel comprised of experts in the medical field and/or medical education experts to evaluate the quality of MCQs created by LLMs; 9 of the study panels comprised of experts in the medical field, 3 of the panels comprised of experts in medical education, and only 2 of the study panels comprised of individuals with qualifications in the relevant medical field and medical education. Eleven (73%) of the studies generated MCQs for undergraduate/postgraduate medicine; two (13%) studies created MCQs for college-level or university-level courses; and only two (13%) studies generated MCQs for other allied health care professions (dentistry, pharmacy).

**Table 1:** Characteristics of included studies.

Author	Year Published	Medical Field, Subspecialty	LLM(s) Evaluated	Number of MCQs Generated by LLM(s)	Control	Evaluation Panel	MCQs Administered to Students
Edwards	2024	Bachelor of Sciences, Pharmaceutical Sciences	ChatGPT 3.5	100	None	Two reviewers (not specified)	No
Ayub	2023	Postgraduate Medicine, Dermatology	ChatGPT 3.5	40	None	Two board-certified dermatologists	No
Mistry	2024	Postgraduate Medicine, Radiology	Llama 2, ChatGPT 4	52, 52	52 human-generated MCQs	Two board-certified radiologists	No
Hwang	2024	College-level Biology	ChatGPT 4	250 (only 150 were evaluated for quality)	None	Two individuals with experience in Biology	No
Laohawetwanit	2024	Postgraduate Medicine, Pathology	ChatGPT 4	80	None	Three board-certified pathologists	Yes
Lotto	2024	Undergraduate/Postgraduate Medicine, Otorhinolaryngology	ChatGPT 3.5	20	None	Senior otolaryngologist and a medical education expert	Yes
Kiyak	2024	Undergraduate Medicine, Pharmacology	ChatGPT 3.5	10 (only 2 administered to students)	None	Expert panel, no details	Yes
Ngo	2024	Undergraduate Medicine, Immunology	ChatGPT 3.5	60	None	Four reviewers, no details	No
Cheung	2023	Undergraduate Medicine	ChatGPT 4	50	50 human-generated MCQs	Five experienced clinicians with involvement in medical education	No
Ahmed	2024	Dentistry	ChatGPT 3.5, Bard 1.0	32, 32	None	Four dental professionals	No
Laupichler	2024	Undergraduate Medicine, Neurophysiology	ChatGPT 3.5	25	25 human-generated MCQs	No quality evaluation by humans	Yes
Rezigalla	2024	Undergraduate Medicine, Anatomy	ChatPDF.com	10	None	Twenty-five medical college staff teaching human anatomy and experienced in medical education	Yes
Schneid	2024	Pharmacy	ChatGPT 3.5	25	25 human-generated MCQs	Expert panel, no details	Yes
Coskun	2024	Undergraduate Medicine, Evidence-Based Medicine	ChatGPT 3.5	15	None	No quality evaluation by humans	Yes
Haynes	2024	Postgraduate Medicine, Internal Medicine	ChatGPT 3.5	9	27 human-generated MCQs	Five attending physicians	Yes

### Risk of bias

Table 2 summarizes the methodological quality of the included studies. All the included studies utilized a quantitative design and methodology; the mean (SD, range) MERSQI score of these studies was 11.82 (1.44, 9.27–15) out of 18. Eight (53%) of the studies utilized a single-group cross-sectional or post-test design, and 7 (47%) of the studies utilized a two-group non-randomized design. Most studies were held at a single institution (73%) and collected objective data (100%) that went beyond a descriptive analysis (87%). 100% of included studies had a data analysis appropriate for study design and type of data. The domain with the poorest scores was the validity of the evaluation instrument. 8 (53%) of the studies were of relatively low quality with scores below 11.6.

**Table 2:** MERSQI scores of included studies.

Study Name	MERSQI Score										
	Study Design (/3)	Institutions (/1.5)	Response Rate (/1.5)	Type of Data (/3)	Internal Structure (/1)	Content (/1)	Relationships to Other Variables (/1)	Appropriateness of Analysis (/1)	Complexity of Analysis (/2)	Outcomes (/3)	Total (/18)
Edwards	1	0.5	N/A	3	1	1	0	1	2	1	11.45
Ayub	1	1	N/A	3	0	1	0	1	1	1	9.82
Mistry	2	0.5	N/A	3	0	1	0	1	2	1	11.45
Hwang	1	0.5	N/A	3	0	1	1	1	2	1	11.45
Laohawetwanit	1	0.5	0.5	3	1	1	0	1	2	2	12
Lotto	2	1.5	0.5	3	1	1	1	1	2	2	15
Kiyak	1	0.5	0.5	3	0	1	0	1	2	1.5	11
Ngo	1	0.5	N/A	3	0	1	0	1	1	1	9.27
Cheung	2	1.5	N/A	3	0	1	0	1	2	1	12.55
Ahmed	2	0.5	N/A	3	0	0	0	1	2	1	10.36
Laupichler	2	0.5	1.5	3	0	1	0	1	2	2	13
Rezigalla	1	0.5	1	3	1	1	1	1	2	2	13.5
Schneid	2	0.5	0.5	3	0	1	0	1	2	2	12
Coskun	1	0.5	1.5	3	0	1	0	1	2	2	12
Haynes	2	1	0.5	3	0	1	0	1	2	2	12.5
Mean Score (SD)	1.47 (0.52)	0.7 (0.37)	0.81 (0.46)	3 (0)	0.27 (0.46)	0.93 (0.26)	0.2 (0.41)	1 (0)	1.87 (0.35)	1.5 (0.5)	11.82 (1.44)

### Outcomes

A summary of the type of analysis performed for each outcome, as well as the LLMs evaluated for each outcome, can be found in Table 3.

**Table 3:** Type of analysis and LLM evaluated for each outcome.

Outcome	Analysis type and LLMs included
Relevance	**Network meta-analysis**: ChatGPT 4, Llama 2, human control **Single-arm analysis:** ChatGPT 3.5, Llama 2 (one study), Chat GPT 4, human control
Clarity	**Network meta-analysis:** ChatGPT 4, Llama 2, human control **Single-arm analysis:** ChatGPT 3.5 (one study), Llama 2 (one study), ChatGPT 4, human control
Distractor Quality	**Network meta-analysis:** ChatGPT 4, Llama 2, human control **Single-arm analysis:** ChatGPT 3.5 (one study), Llama 2 (one study), ChatGPT 4, human control
Accuracy	**Pairwise meta-analysis:** ChatGPT 3.5, Bard **Single-arm analysis:** ChatGPT 3.5, Bard (one study)
Item Difficulty Index	**Pairwise meta-analysis:** ChatGPT 3.5, Bard **Single-arm analysis:** ChatGPT 4 (one study), ChatGPT 3.5, ChatPDF.com (one study), human control
Item Discrimination Analysis – Item Discrimination Index	**Pairwise meta-analysis:** ChatGPT 3.5, human control **Single-arm analysis:** ChatGPT 4 (one study), ChatGPT 3.5, ChatPDF.com (one study), human control
Item Discrimination Analysis – Point Biserial Correlation	**Pairwise meta-analysis:** ChatGPT 3.5, human control **Single-arm analysis:** ChatGPT 3.5, human control

#### Relevance.

Five studies assessed the relevance of MCQs on a Likert scale utilizing an expert panel. Two studies evaluated two or more interventions and were included in the NMA. The remaining three investigations were single-arm studies, and their results were solely included in the single-arm analysis. LLMs assessed included ChatGPT 3.5, ChatGPT 4 and Llama 2, with a human control arm present.

**Fig 2:**
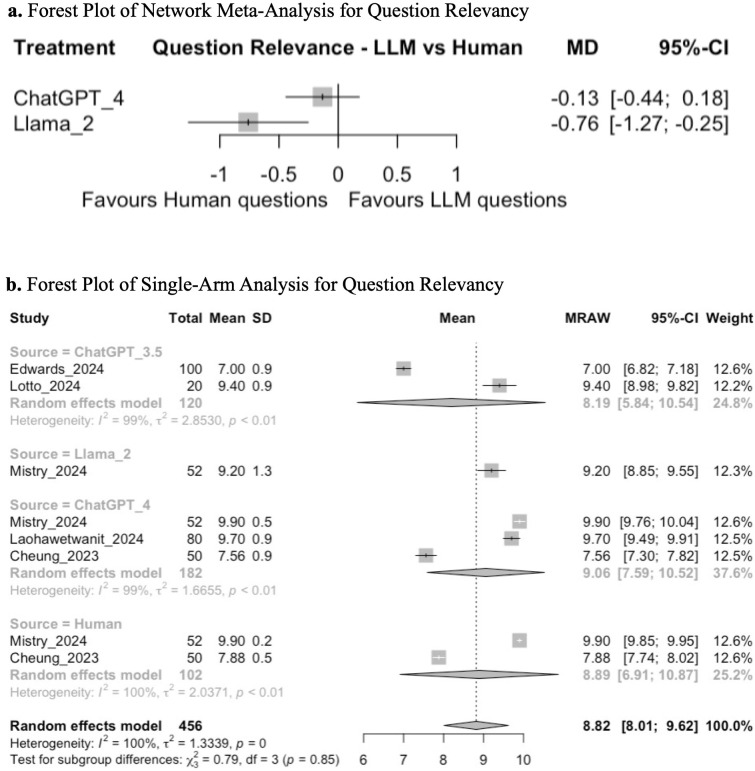
**(a)** Forest plot of network meta-analysis for question relevancy. **(b)** Forest plot of single-arm analysis for question relevancy.

Network meta-analysis between ChatGPT 4, Llama 2, and humans demonstrated that there was no significant difference in the relevance of questions generated by ChatGPT 4 compared to humans (MD −0.13; 95% CI: −0.44, 0.18; GRADE: VERY LOW), but MCQs generated by Llama 2 may be less relevant than those created by humans (MD −0.76; 95% CI: −1.27, −0.25; GRADE: VERY LOW) (Fig 2a, S1 Fig).

Single-arm analysis demonstrated a mean pooled transformed score of 8.82 (95% CI: 8.01, 9.62; GRADE: VERY LOW; subgroup analysis between ChatGPT 3.5, ChatGPT 4, Llama 2, and human-generated questions did not demonstrate a significant difference in question relevancy (chi-squared: 0.79; df = 3; *p* = 0.85) (Fig 2b).

#### Clarity.

Four studies assessed the clarity of MCQs on a Likert scale via an expert panel. Two studies evaluated two or more interventions and were included in the NMA. The remaining two investigations were single-arm studies, and their results were solely included in the single-arm analysis. LLMs assessed included ChatGPT 3.5, ChatGPT 4, and Llama 2, with a human control arm present.

**Fig 3:**
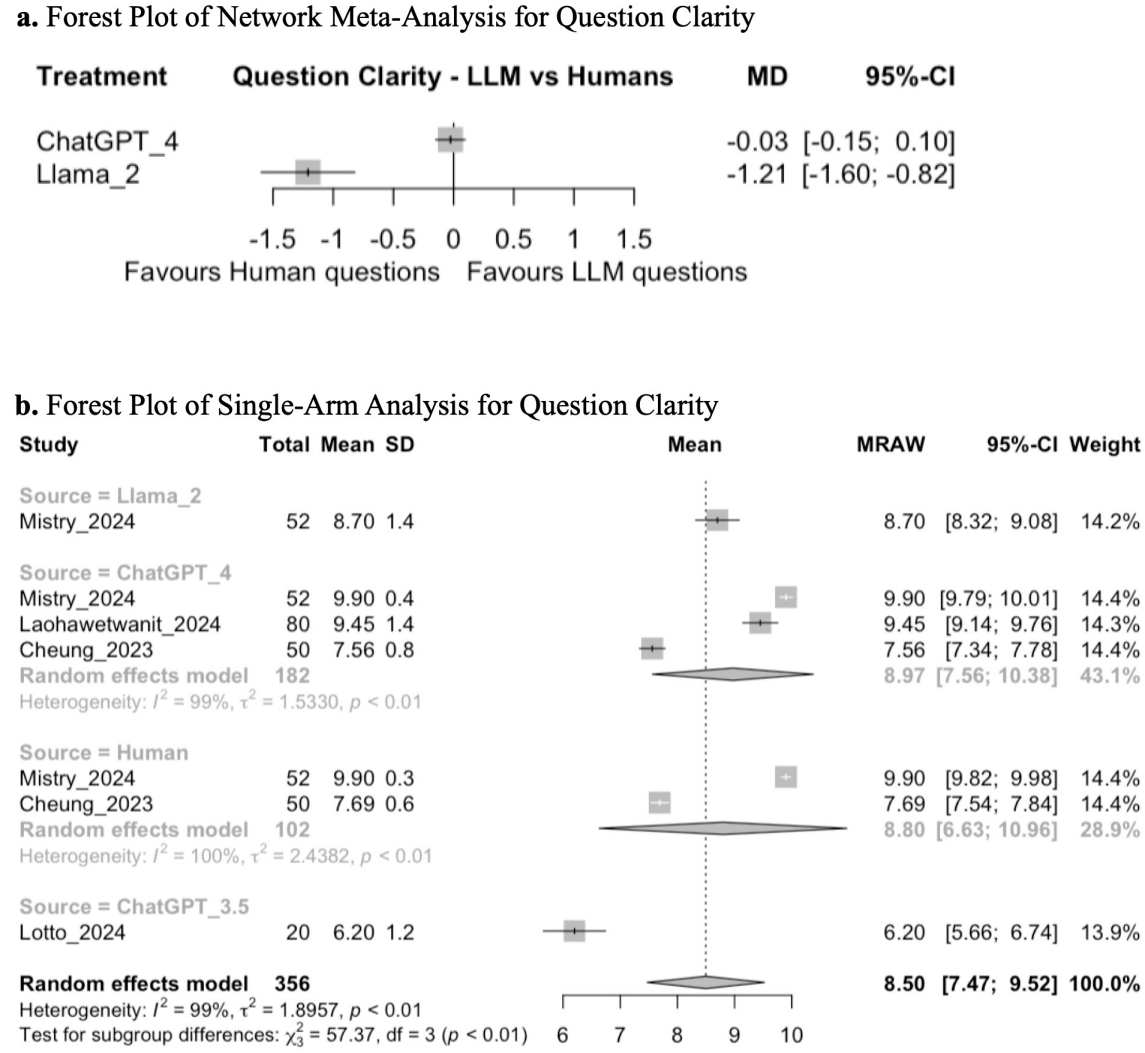
**(a)** Forest plot of network meta-analysis for question clarity. **(b)** Forest plot of single-arm analysis for question clarity.

NMA evaluating ChatGPT 4, Llama 2, and humans demonstrated that there was no significant difference in the clarity of questions generated by ChatGPT 4 compared to humans (MD −0.03; 95% CI: −0.15, 0.10; GRADE: VERY LOW), but MCQs generated by Llama 2 may be less clear than those created by humans (MD −1.21; 95% CI: −1.60, −0.82; GRADE: VERY LOW) (Fig 3a, S2 Fig). Subsequent single-arm analysis demonstrated a mean pooled score of 8.5 (95% CI: 7.47, 9.52; GRADE: VERY LOW). Tests for subgroup analysis between ChatGPT 3.5, ChatGPT 4, Llama 2, and human-generated questions demonstrated a significant difference in question clarity (chi-squared: 57.37; df = 3, *p* < 0.01) (Fig 3b). Exploratory post-hoc t-tests demonstrated that ChatGPT 3.5 had lower clarity scores than ChatGPT 4 (p < 0.001), Llama 2 (p < 0.001), and humans (p = 0.022).

#### Distractor quality.

Four studies assessed the quality of MCQ distractors on a Likert scale via an expert panel. Two studies evaluated two or more interventions and were included in the NMA. The remaining two investigations were single-arm studies, and their results were solely included in the single-arm analysis. LLMs assessed included ChatGPT 3.5, ChatGPT 4, and Llama 2, with a human control arm present.

**Fig 4:**
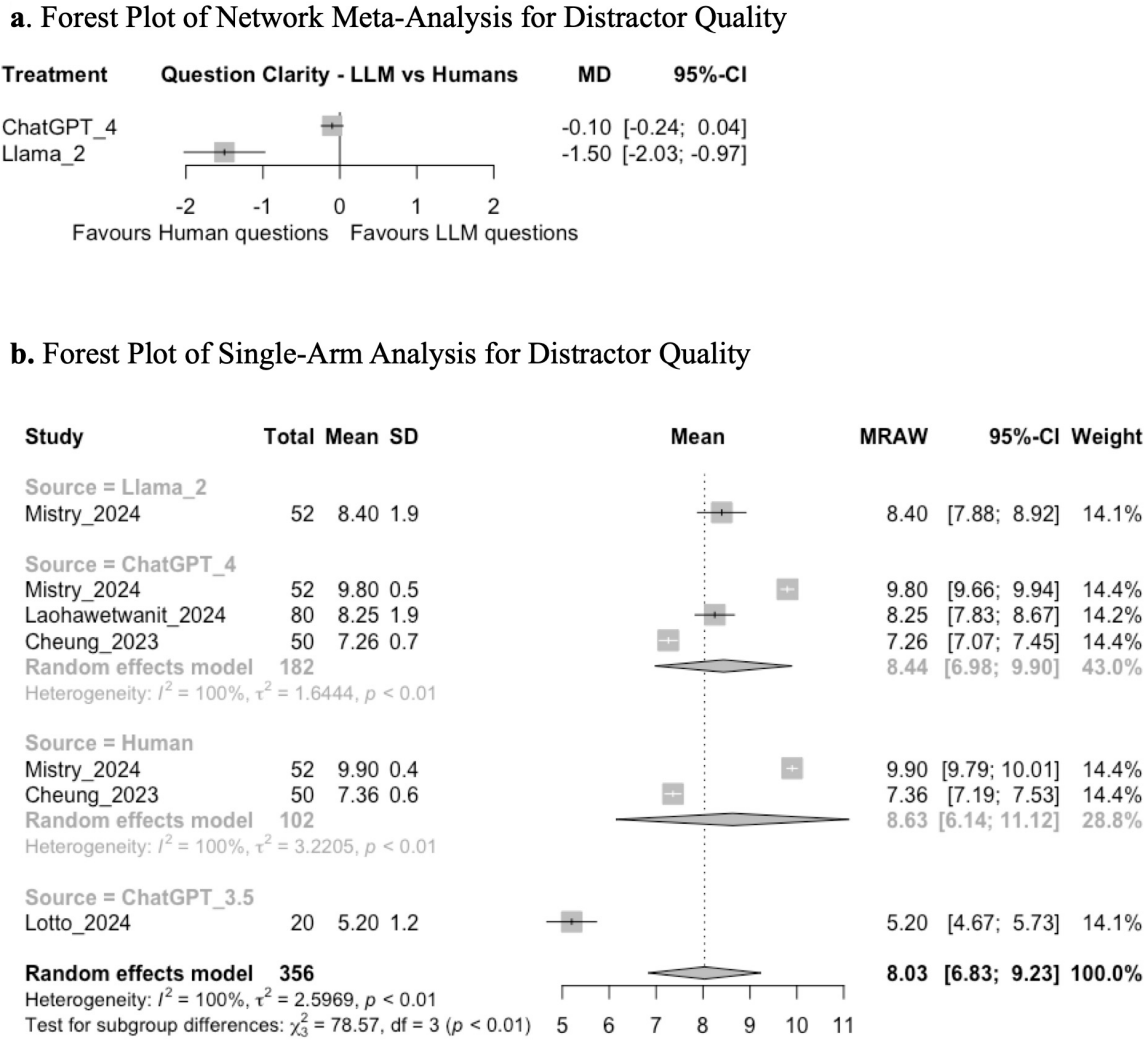
**(a)** Forest plot of network meta-analysis for distractor quality. **(b)** Forest plot of single-arm analysis for distractor quality.

Network meta-analysis between ChatGPT 4, Llama 2, and humans demonstrated that there was no significant difference in the quality of distractors generated by ChatGPT 4 compared to humans (MD −0.10; 95% CI: −0.24, 0.04; GRADE: VERY LOW), but MCQs generated by Llama 2 may have lower quality of distractors than those created by humans (MD −1.50; 95% CI: −2.03, −0.97; GRADE: VERY LOW) (Fig 4a, S3 Fig). Subsequent single-arm analysis demonstrated a mean pooled score of 8.03 (95% CI: 6.83, 9.23; GRADE: VERY LOW). Tests for subgroup analysis between ChatGPT 3.5, ChatGPT 4, Llama 2, and human-generated questions demonstrated a significant difference in distractor quality (chi-squared: 78.57; df = 3, *p* < 0.01) (Fig 4b). Exploratory post-hoc t-tests demonstrated that ChatGPT 3.5 had lower distractor quality scores than ChatGPT 4 (p < 0.001), Llama 2 (p < 0.001), and humans (p = 0.008).

#### Accuracy.

Four studies assessed the accuracy of MCQs by utilizing an expert panel to determine the % correct multiple choice questions. One study compared two interventions and was evaluated in a pairwise analysis, the remaining three studies were single arm analyses. LLMs assessed included ChatGPT 3.5 and Bard 1.0, with no human control arm.

**Fig 5:**
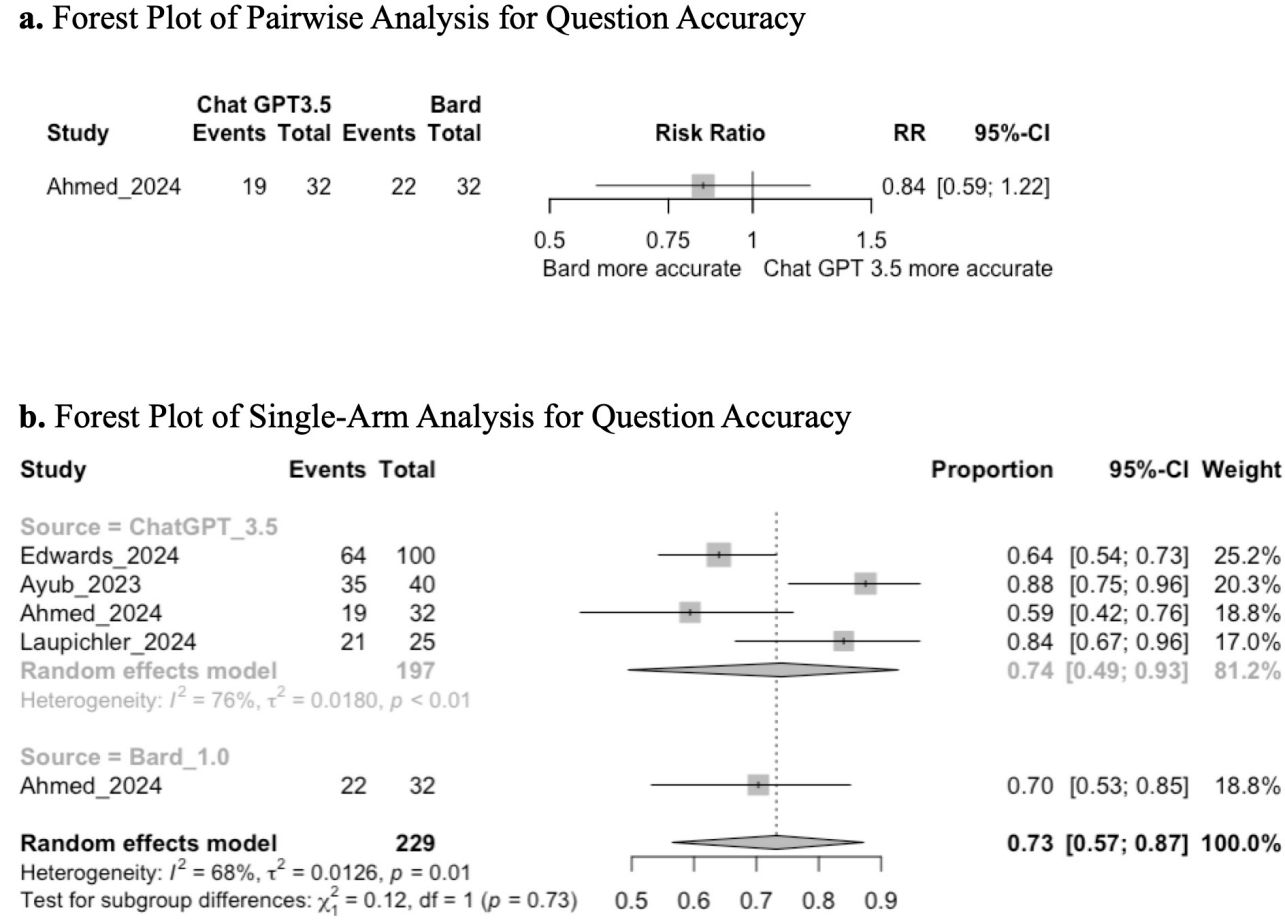
**(a)** Forest plot of pairwise analysis for question accuracy. **(b)** Forest plot of single-arm analysis for question accuracy.

Pairwise analysis demonstrated that there was no significant difference in accuracy between Chat GPT3.5 and Bard (RR: 0.84; 95%CI: 0.59 to 1.22; GRADE: Very Low) (Fig 5a).

Single-arm analysis demonstrated a mean pooled score of 0.73 (95% CI: 0.57, 0.87; GRADE: VERY LOW); subgroup analysis between ChatGPT 3.5 and Bard-generated questions did not demonstrate a significant difference in question accuracy (chi-squared: 0.12; df = 1; *p* = 0.73) (Fig 5b).

#### Item difficulty analysis.

Seven studies performed an item difficulty analysis by assessing the difficulty index of MCQs. Three studies compared Chat GPT 3.5 to a human control and were included in the pairwise analysis. LLMs assessed included ChatGPT 3.5, ChatGPT 4, and ChatPDF.com, with a human control arm present.

**Fig 6:**
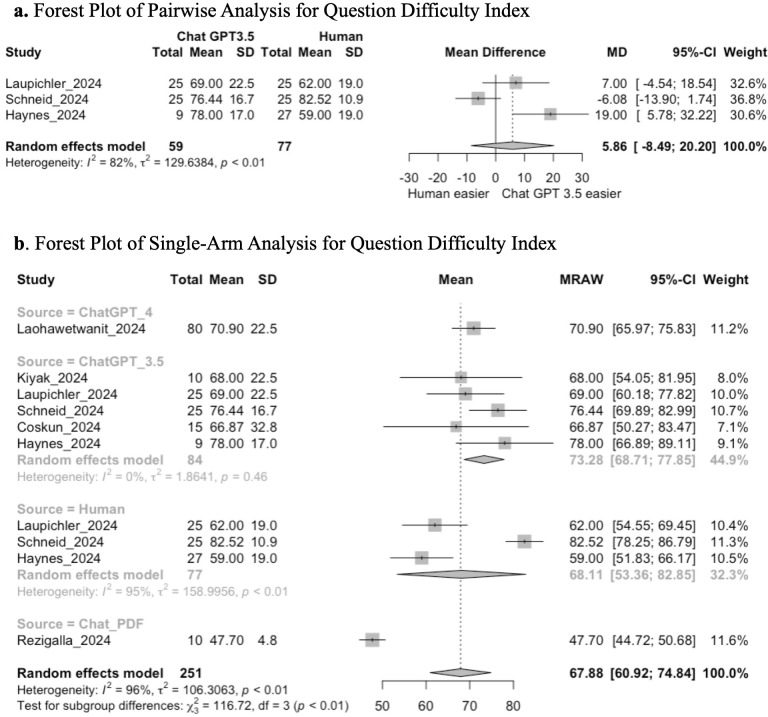
**(a)** Forest plot of pairwise analysis for question difficulty index. **(b)** Forest plot of single-arm analysis for question difficulty index.

Pairwise analysis between studies comparing ChatGPT 3.5 and humans demonstrated that there is no significant difference in the difficulty index (MD 5.86; 95% CI: −8.49, 20.20; GRADE: VERY LOW) (Fig 6a).

Single-arm analysis demonstrated a mean pooled score of 67.88 (95% CI: 60.92, 74.84; GRADE: VERY LOW). Tests for subgroup analysis between ChatGPT 3.5, ChatGPT 4, ChatPDF.com, and human-generated questions demonstrated a significant difference in the difficulty index (chi-squared: 116.72; df = 3, *p* < 0.01) (Fig 6b). Exploratory post-hoc t-tests demonstrated that ChatPDF.com had a lower difficulty index than ChatGPT 3.5 (p < 0.001), ChatGPT 4 (p = 0.010), and humans (p < 0.001).

#### Item discrimination analysis – item discrimination index.

Five studies performed an item discrimination analysis of MCQs by assessing the average item discrimination index. Two studies compared both Chat GPT 3.5 to human controls and were included in the pairwise meta-analysis. LLMs assessed included ChatGPT 3.5, ChatGPT 4, and ChatPDF.com, with a human control arm present.

**Fig 7:**
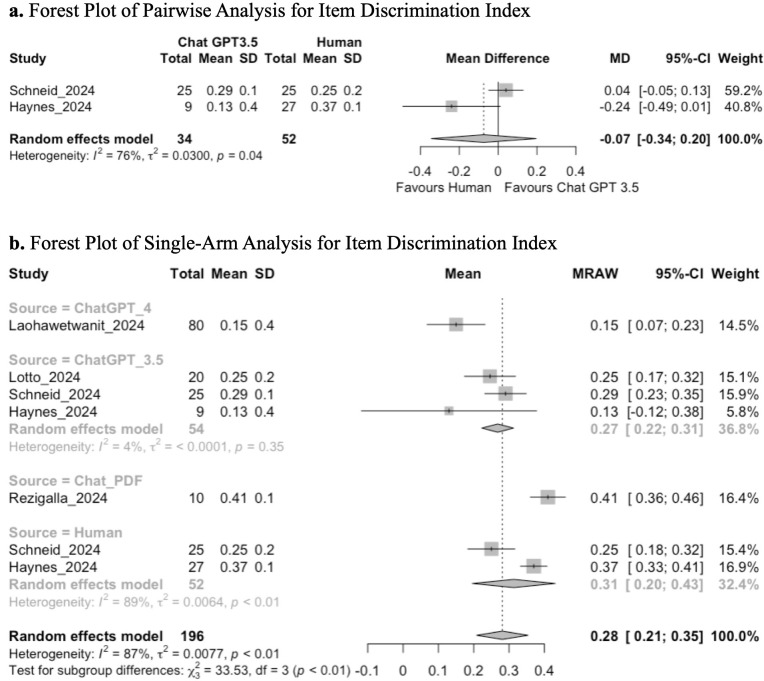
**(a)** Forest plot of pairwise analysis for item discrimination index. **(b)** Forest plot of single-arm analysis for item discrimination index.

Pairwise analysis between ChatGPT 3.5 and humans demonstrated that there is no significant difference in the average item discrimination index (MD −0.07; 95% CI: −0.34, 0.20; GRADE: VERY LOW) (Fig 7a).

Single-arm analysis demonstrated a mean pooled score of 0.28 (95% CI: 0.21, 0.35; GRADE: VERY LOW). Tests for subgroup analysis between ChatGPT 3.5, ChatGPT 4, ChatPDF.com, and human-generated questions demonstrated a significant difference in the average item discrimination index (chi-squared: 33.53; df = 3, *p* < 0.01) (Fig 7b). Exploratory post-hoc t-tests demonstrated that ChatPDF.com had a higher average item discrimination index than ChatGPT 3.5 (p < 0.001) and ChatGPT 4 (p < 0.001). Additionally, questions produced by Chat GPT4.0 had a lower item discrimination analysis compared with humans (p = 0.025), and Chat GPT 3.5 (p = 0.010).

#### Item discrimination analysis – point biserial correlation.

Four studies performed an item discrimination analysis of MCQs by assessing the point biserial correlation. Two studies compared both Chat GPT 3.5 to human controls and were included in the pairwise meta-analysis. The LLMs assessed were ChatGPT 3.5, and human generated questions.

**Fig 8:**
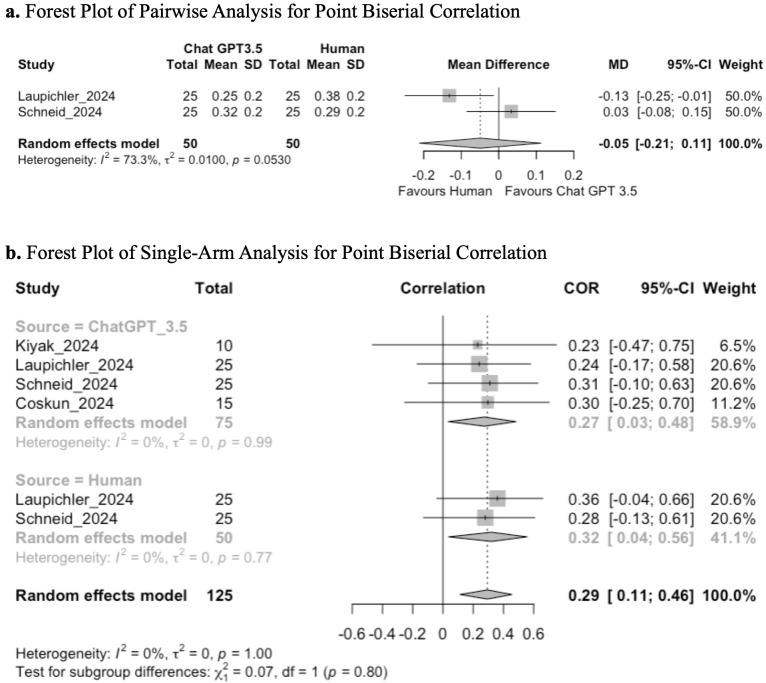
**(a)** Forest plot of pairwise analysis for point biserial correlation. **(b)** Forest plot of single-arm analysis for point biserial correlation.

Pairwise meta-analysis demonstrated no significant difference in scores between the Chat GPT 3.5 and human generated questions (MD: −0.05; 95% CI: −0.21 to 0.11; GRADE: VERY LOW) (Fig 8a).

Single-arm analysis demonstrated a mean pooled score of 0.29 (95% CI: 0.11, 0.46; GRADE: VERY LOW). There was no significant difference in the subgroup analysis (chi-squared = 0.07; p = 0.80) (Fig 8b).

### Certainty of evidence

The certainty of evidence for all outcomes was rated as very low. Outcomes were most frequently downgraded due to serious concerns regarding indirectness, imprecision, or risk of bias (S2 Table).

## Discussion

Utilizing LLMs to generate valid, reliable, and accurate multiple-choice questions has the potential to change the field of medical education. In this systematic review and network meta-analysis, the quality of multiple-choice questions generated by five LLMs for undergraduate and postgraduate medical examinations was evaluated based on seven pre-specified outcomes. To our knowledge, this is the first systematic review on this topic that was able to perform a statistical analysis of the data. The results demonstrate that the quality of multiple-choice questions generated by ChatGPT 4 and ChatPDF.com may be comparable to those generated by humans, while questions generated by ChatGPT 3.5 and Llama 2 may be of worse quality than those generated by humans; however, the certainty of evidence is very low across all outcomes, and significantly more research is needed.

There may be no significant difference between the quality of MCQs generated by ChatGPT 4 and humans with regards to qualitative measures of question quality (question relevance, question clarity, and distractor quality); this is of very low certainty of evidence. However, only one study evaluated the quality of ChatGPT 4 on more quantitative measures such as the difficulty index, item discrimination index, and point biserial correlation, and there were no studies that directly compared the quality of MCQs written by ChatGPT 4 to those written by humans on these quantitative indices. To delineate whether the quality of MCQs generated by ChatGPT 4 are similar in quality to those written by humans, head-to-head studies need to be conducted that are adequately controlled and administered to students to assess quantitative measures of quality.

Multiple choice questions generated by ChatGPT 3.5 may be of worse quality than those generated by humans, and previous studies have shown that instructions written by ChatGPT 3.5 are less understandable than those written by humans [43]. In our analysis, ChatGPT 3.5 performed worse than ChatGPT 4 and Llama 2 with regards to question clarity and distractor quality. Given that NMA suggests the quality of MCQs generated by Llama 2 is worse than those created by humans with regards to question clarity and distractor quality, we hypothesize that ChatGPT 3.5 may also perform worse than humans on these indices. There was no significant difference in the difficulty index, item discrimination index, or point biserial correlation between ChatGPT 3.5 and human; however, the trend suggests that questions written by humans are more difficult and more discriminative between high- and low-performing students than those written by ChatGPT 3.5. The literature suggests that easy MCQs have a difficulty index of 70% and greater, and moderately difficult MCQs have a difficulty index of less than 70% [44]; in our study, MCQs written by ChatGPT 3.5 had a difficulty index of 73.28% whereas those written by humans had a difficulty index of 68.11%. Additionally, an ideal item discrimination index and point biserial correlation for large scale standardized tests is 0.3 [34]; ChatGPT 3.5 had an item discrimination index and point biserial correlation less than 0.3, whereas humas were above the threshold of 0.3 for both indices. The quality of this evidence is very low, and studies comparing the quality of questions written by ChatGPT 3.5 to humans in a systematic fashion is needed.

Finally, ChatPDF.com may create MCQs that are more difficult and have a higher ability to discriminate between high- and low-scoring students than ChatGPT 3.5 and ChatGPT 4. However, this is of very low certainty of evidence, and there was only one study that evaluated ChatPDF.com with no control data. Further investigations evaluating this LLM is required.

It is vital to highlight that the quality of multiple-choice questions generated by LLMs can be negatively affected in a multitude of ways, including the design of the prompt provided to the LLM as well as hallucination [12,45]. In practice, hallucination can lead to the generation of inaccurate material that may appears plausible on the surface, disrupt difficulty calibration by masking what the model knows, and undermine discrimination by making questions/explanations inconsistent and unreliable. Human experts within the field of study are required to routinely fact-check the question stem and answer key generated by AI to ensure that content is accurate, coherent, unbiased, and able to accurately discriminate between students [46]. It may be beneficial for educators to have formal training in prompt design, as well as in critically analyzing output from LLM to ensure content accuracy. Constraining the LLM to specific resources, such as course textbooks or instructor-provided resources, can be helpful in ensuring the content is generated from reputable and reliable resources. Educators should consider piloting LLM-generated MCQs on a small subset of students prior to integrating questions into formal examinations. Of the LLMs evaluated in this network meta-analysis, ChatGPT 4 is potentially the best resource for educators to generate drafts of MCQs, although quality review of the generated content is required, and the performance of questions created by ChatGPT 4 on quantitative indices is unknown. Other LLMs, such as ChatGPT 3.5 or Llama 2, are less likely to produce accurate and unbiased content, given their worse performance on qualitative and quantitative measures of question quality. Finally, while our study suggests that ChatPDF.com may produce more difficult and discriminating questions, this signal requires further study and evaluation to truly delineate the quality of the questions.

Our review has several limitations. There was variability in how MCQ quality was defined and measured across studies. Only a minority of studies were comparative; the remainder relied on single-group cross-sectional or non-randomized two-group designs, usually from a single institution and with small sample sizes. Such characteristics underpin the “very low” GRADE ratings. There are no published randomized control trials evaluating the quality of MCQs written by LLMs to those written by humans. Two-thirds of the studies included in our review did not perform head-to-head studies evaluating the quality of LLM-generated MCQs to those written by humans. Approximately half of the studies did not administer the MCQs to students; thus, the conclusions regarding the ability of LLMs to generate high-quality MCQs are drawn from a relatively small and contextually heterogenous set of studies. The certainty of evidence is very low and was limited by risk of bias within the studies, inconsistency, and imprecision; this was particularly evident with limited data to perform the network meta-analysis. Although inter-rater reliability among experts may be adequate, the limited validity evidence warrants restraint in inferring meaningful distinctions from variations in Likert-scale ratings of clarity, relevance, and distractor quality. One notable limitation is that most included studies focused on undergraduate and postgraduate medicine, with relatively few conducted in other allied health care professions. The extent to which our findings generalize beyond the field of medicine remains unclear. Finally, given the rapid evolution of LLMs, future or novel models may not align with the patterns observed in this systematic review and network meta-analysis. Nevertheless, the findings provide meaningful insights and highlight the need for further high-quality research across a broader range of health care fields to better understand the role of LLM-generated MCQs in medical education.

## Conclusion

To our knowledge, this is the first systematic review to apply network meta-analysis comparing all eligible studies evaluating the quality of MCQs generated by LLMs in the field of medical education. The results suggest that ChatGPT 4 may create MCQs of similar quality to those written by humans on certain qualitative outcomes. Questions generated by Llama 2 and ChatGPT 3.5 perform worse on key outcomes that pertain to question quality, whereas those generated by ChatPDF.com may perform better on key quantitative outcomes. To address the limitations of this analysis, further studies are needed that directly compare the above LLMs with human-generated MCQs in a systematic manner, administering these MCQs to students across varied allied health care fields to evaluate whether LLMs can be effectively used to generate examination questions in health care professions education. Given the rapid evolution of LLM capabilities, our findings provide a timely contribution to the growing literature, offering a contemporary benchmark for educators and researchers to inform the integration of these tools into medical assessment design.

## Supporting information

S1 Fig Network plot for question relevancy. (PDF)

S2 Fig Network plot for question clarity. (PDF)

S3 Fig Network plot for distractor quality. (PDF)

S1 Table PRISMA 2020 checklist. (PDF)

S2 Table Summary of GRADE ratings for each outcome. (PDF)

S1 File Search strategy. (PDF)
