# Supplementary figures and images for: The use of large language models in generating multiple choice questions for health professions education: A systematic review and network meta-analysis
Source: PLoS One. 2026 Jan 2;21(1):e0340277. doi: 10.1371/journal.pone.0340277 (PMC12758716; doi:10.1371/journal.pone.0340277)

**S1 Fig.** Network Plot for Question Relevancy

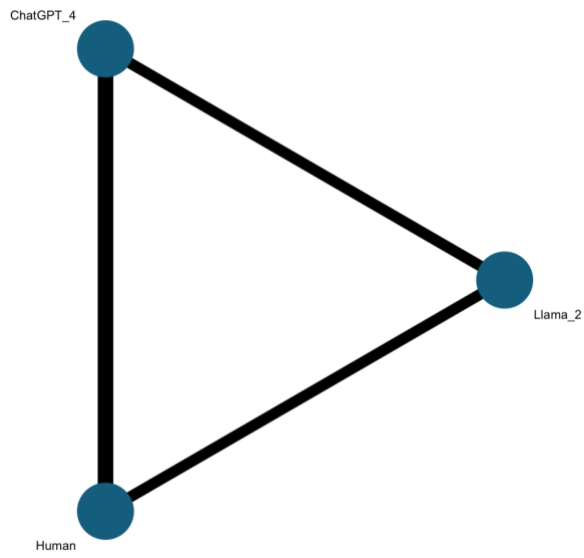

Supplement: S1 Fig — (PDF) [file pone.0340277.s001.pdf]

**S2 Fig.** Network Plot for Question Clarity

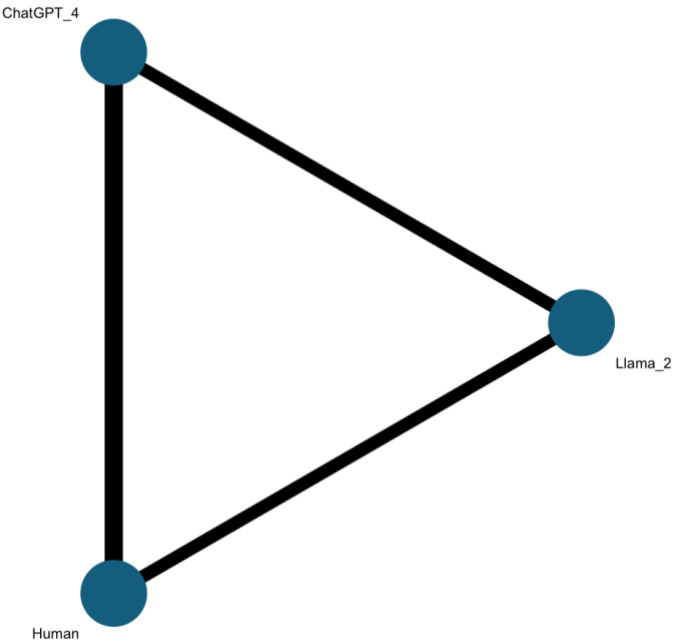

Supplement: S2 Fig — (PDF) [file pone.0340277.s002.pdf]

**S3 Fig.** Network Plot for Distractor Quality

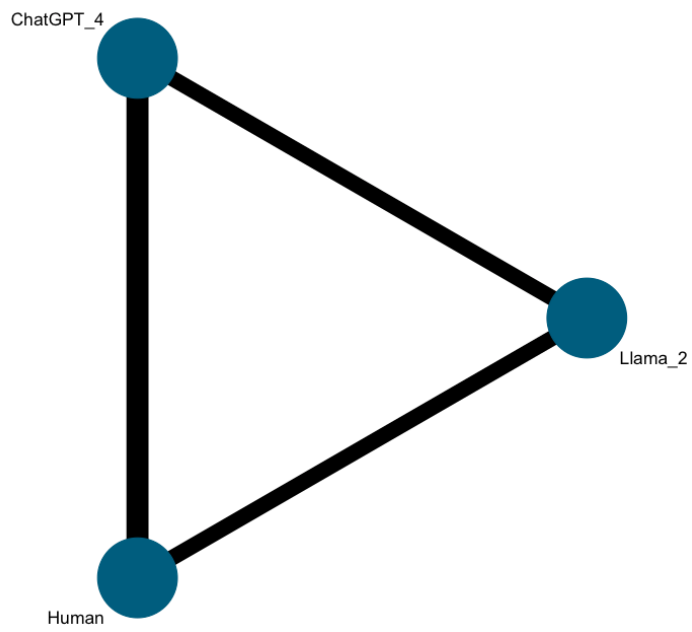

Supplement: S3 Fig — (PDF) [file pone.0340277.s003.pdf]
