# Supplementary material for: The use of large language models in generating multiple choice questions for health professions education: A systematic review and network meta-analysis
Source: PLoS One. 2026 Jan 2;21(1):e0340277. doi: 10.1371/journal.pone.0340277 (PMC12758716; doi:10.1371/journal.pone.0340277)
Supplement: S2 Table — (PDF) [file pone.0340277.s005.pdf]

S2 Table. Summary of GRADE ratings for each outcome

Certainty Assessment						№ of questions	Relative Effect	Certainty of Evidence
№ of studies	Risk of bias	Inconsistency	Indirectness	Imprecision	Publication bias			

Relevancy

Single-Arm Analysis

5 studies	Not serious	Serious[b]	Not serious	Not serious	Not serious	456	MRAW 8.82 (8.01 to 9.62)	⊕◯◯◯ Very Low

Network Meta-Analysis

Comparison	No of studies	Risk of Bias	Inconsistency	Indirectness	Publication Bias	Intransitivity	Imprecision	Incoherence	No of Questions	Relative Effect (MD)	Certainty of Evidence
Chat GPT 4 vs. Human	2	Some concerns (-0.5)	Not serious	Not serious	Not serious	Not Serious	Serious (-1)	Not serious	256	-0.13 (-0.44 to 0.18)	⊕◯◯◯ Very Low
Llama 2 vs. Human	2	Some concerns (-0.5)	Not serious	Not serious	Not serious	Not serious	Serious (-1)	Not serious	256	-0.76 (-1.27 to -0.25)	⊕◯◯◯ Very Low
Chat GPT 4 vs Llama 2	2	Some concerns (-0.5)	Not serious	Not serious	Not serious	Not serious	Serious (-1)	Not serious	256	0.63 (0.11 to 1.14)	⊕◯◯◯ Very Low

Certainty Assessment						№ of questions	Relative Effect	Certainty of Evidence
№ of studies	Risk of bias	Inconsistency	Indirectness	Imprecision	Publication bias			

Clarity

Single-Arm Analysis

4 studies	Not serious	Serious[b]	Not serious	Not serious	Not serious	356	MRAW 8.50 (7.47 to 9.52)	⊕◯◯◯ Very Low

Network Meta-Analysis

Comparison	No of studies	Risk of Bias	Inconsistency	Indirectness	Publication Bias	Intransitivity	Imprecision	Incoherence	No of Questions	Relative Effect (MD)	Certainty of Evidence
Chat GPT 4 vs. Human	2	Some concerns (-0.5)	Not serious	Not serious	Not serious	Not Serious	Serious Concerns (-1)	Not serious	256	-0.03 (-0.15 to 0.10)	⊕◯◯◯ Very Low
Llama 2 vs. Human	2	Some concerns (-0.5)	Not serious	Not serious	Not serious	Not serious	Serious Concerns (-1)	Not serious	256	-1.21 (-1.60 to -0.82)	⊕◯◯◯ Very Low
Chat GPT 4 vs Llama 2	2	Some concerns (-0.5)	Not serious	Not serious	Not serious	Not serious	Serious Concerns (-1)	Not serious	256	1.18 (0.79 to 1.58)	⊕◯◯◯ Very Low

Certainty Assessment						№ of questions	Relative Effect	Certainty of Evidence
№ of studies	Risk of bias	Inconsistency	Indirectness	Imprecision	Publication bias			

Distractor Quality

Single Arm-Analysis

Certainty Assessment						№ of questions	Relative Effect	Certainty of Evidence
№ of studies	Risk of bias	Inconsistency	Indirectness	Imprecision	Publication bias			

Distractor Quality

4 studies	Not serious	Serious[b]	Not serious	Serious[c]	Not serious	356	MRAW 8.03 (6.83 to 9.23)	⊕◯◯◯ Very Low

Network Meta-Analysis

Comparison	No of studies	Risk of Bias	Inconsistency	Indirectness	Publication Bias	Intransitivity	Imprecision	Incoherence	No of Questions	Relative Effect (MD)	Certainty of Evidence
Chat GPT 4 vs. Human	2	Some concerns (-0.5)	Not serious	Not serious	Not serious	Not Serious	Serious Concerns (-1)	Not serious	256	-0.10 (-0.24 to 0.04)	⊕◯◯◯ Very Low
Llama 2 vs. Human	2	Some concerns (-0.5)	Not serious	Not serious	Not serious	Not serious	Serious Concerns (-1)	Not serious	256	-1.50 (-2.03 to -0.97)	⊕◯◯◯ Very Low
Chat GPT 4 vs Llama 2	2	Some concerns (-0.5)	Not serious	Not serious	Not serious	Not serious	Serious Concerns (-1)	Not serious	256	1.50 (0.97 to 2.03)	⊕◯◯◯ Very Low

Certainty Assessment						№ of questions	Relative Effect	Certainty of Evidence
№ of studies	Risk of bias	Inconsistency	Indirectness	Imprecision	Publication bias			

Accuracy

Single Arm-Analysis

4 studies	Serious[a]	Serious[b]	Not serious	Serious[c]	Not serious	229	MRAW 0.73 (0.57 to 0.87)	⊕◯◯◯ Very Low

Pairwise Analysis

1 study	Serious	N/A	Not serious	Serious[c]	Not serious	64	RR: 0.84 (0.59 to 1.22)	⊕◯◯◯ Very Low

Certainty Assessment						№ of questions	Relative Effect	Certainty of Evidence
№ of studies	Risk of bias	Inconsistency	Indirectness	Imprecision	Publication bias			

Item Difficulty Analysis – Difficulty Index

Single-Arm Analysis

7 studies	Not serious	Serious[b]	Not serious	Not serious	Not serious	251	MRAW 67.88 (60.92 to 74.84)	⊕◯◯◯ Very Low

Pairwise Analysis

3 studies	Not serious	Serious[b]	Not serious	Serious[c]	Not serious	136	MRAW 5.86 (-8.49 to 20.20)	⊕◯◯◯ Very Low

Certainty Assessment						№ of questions	Relative Effect	Certainty of Evidence
№ of studies	Risk of bias	Inconsistency	Indirectness	Imprecision	Publication bias			
**Item Discrimination Analysis – Item Discrimination Index**								
**Single-Arm Analysis**								
3 studies	Not serious	Serious[b]	Not serious	Serious[c]	Not serious	196	MRAW 0.28 (0.21 to 0.35)	⊕◯◯◯ Very Low
**Pairwise Analysis**								
2 studies	Not serious	Not serious	Not serious	Serious[c]	Not serious	86	MRAW -0.07 (0.34 to 0.20)	⊕◯◯◯ Very Low

Certainty Assessment						№ of questions	Relative Effect	Certainty of Evidence
№ of studies	Risk of bias	Inconsistency	Indirectness	Imprecision	Publication bias			
**Item Discrimination Analysis – Point Biserial Correlation**								
**Single-Arm Analysis**								
4 studies	Not serious	Not serious	Not serious	Serious[c]	Not serious	125	MRAW 0.29 (0.11 to 0.46)	⊕◯◯◯ Very Low
**Pairwise Analysis**								
2 studies	Not serious	Serious	Not serious	Serious	Not serious	100	MD: -0.05 (-0.21 to 0.11)	⊕◯◯◯ Very Low

Abbreviations:
CI: Confidence intervals

Rationale:

a. There were serious concerns for risk of bias across the included studies
b. Effect estimates were heterogenous across the included studies
c. Pooled effect estimate crosses the boundary of what is likely clinically significant. Given the lack of an established minimally important differences for this outcome, extrapolating from guidance from the GRADE handbook, the certainty was downgraded if the difference between the upper and lower limit of the 95% confidence interval crossed a threshold of 25% of the pooled effect estimate.[1]

References:

1. GRADE handbook. Accessed January 10, 2025. https://gdt.gradepro.org/app/handbook/handbook.html
