# Supplementary material for: The use of large language models in generating multiple choice questions for health professions education: A systematic review and network meta-analysis
Source: PLoS One. 2026 Jan 2;21(1):e0340277. doi: 10.1371/journal.pone.0340277 (PMC12758716; doi:10.1371/journal.pone.0340277)
Supplement: S1 File — (PDF) [file pone.0340277.s006.pdf]

S1 File: Search strategy

MEDLINE:
exp Artificial Intelligence/ or (AI or "artificial intelligence" or AIVI or "classification algorithm*" or "computer heuristic*" or "convolutional network*" or DALL-E or "decision support system*" or "decision tree" or DeepAI or "deep learning" or "data science" or "feature detection" or "generative pre-trained transformer" or "generative pretrained transformer" or Invideo or "language learning model*" or "large language model*" or "learning algorithm*" or "machine learning" or (Markov adj3 model*) or Midjourney or ((multifactor* or multicriteria) adj3 ("decision analysis" or "decision making")) or "natural language process*" or "nearest neighbo*" or "neural network*" or "outlier detection" or "pattern recognition" or Perplexity or "probability tree" or "random forest" or "representation learning" or Runway AI or Runway Gen-1 or "Stable Diffusion" or "support vector machine*" or "transfer learning" or "Bing chat" or ChatGPT* or "Chat GPT" or “Chat-GPT” or “GPT” or "Google* Bard" or “Bard” or "Google* Gemini" or "IBM Watson" or "Microsoft* Bing" or "Microsoft* Copilot" or “LLaMA” or OpenAI or "Open AI" or PathAI or "Path AI").mp.
AND (“MCQ*” or “multiple choice question*”).mp.

EMBASE:
exp artificial intelligence/ or exp deep learning/ or exp machine learning/ or (AI or "artificial intelligence" or AIVI or "classification algorithm*" or "computer heuristic*" or "convolutional network*" or DALL-E or "decision support system*" or "decision tree" or DeepAI or "deep learning" or "data science" or "feature detection" or "generative pre-trained transformer" or "generative pretrained transformer" or Invideo or "language learning model*" or "large language model*" or "learning algorithm*" or "machine learning" or (Markov adj3 model*) or Midjourney or ((multifactor* or multicriteria) adj3 ("decision analysis" or "decision making")) or "natural language process*" or "nearest neighbo*" or "neural network*" or "outlier detection" or "pattern recognition" or Perplexity or "probability tree" or "random forest" or "representation learning" or Runway AI or Runway Gen-1 or "Stable Diffusion" or "support vector machine*" or "transfer learning" or "Bing chat" or ChatGPT* or "Chat GPT" or “Chat-GPT” or “GPT” or "Google* Bard" or “Bard” or "Google* Gemini" or "IBM Watson" or "Microsoft* Bing" or "Microsoft* Copilot" or “LLaMA” or OpenAI or "Open AI" or PathAI or "Path AI").mp.
AND (“MCQ*” or “multiple choice question*”).mp.

Scopus:
TITLE-ABS-KEY (ai OR "artificial intelligence" OR aivi OR "classification algorithm*" OR "computer heuristic*" OR "convolutional network*" OR dall-e OR "decision support system*" OR "decision tree" OR deepai OR "deep learning" OR "data science" OR "feature detection" OR "generative pre-trained transformer" OR "generative pretrained transformer" OR invideo OR "language learning model*" OR "large language model*" OR "learning algorithm*" OR "machine learning" OR (markov W/3 model*) OR midjourney OR ((multifactor* OR multicriteria) W/3 ("decision analysis" OR "decision making")) OR "natural language process*" OR "nearest neighbo*" OR "neural network*" OR "outlier detection" OR "pattern recognition" OR perplexity OR "probability tree" OR "random forest" OR "representation learning" OR runway AND ai OR runway AND gen-1 OR "Stable Diffusion" OR "support vector machine*" OR "transfer learning" OR "Bing chat" OR chatgpt* OR "Chat GPT" OR

"Chat-GPT" OR "GPT" OR "Google* Bard" OR "Bard" OR "Google* Gemini" OR "IBM Watson" OR "Microsoft* Bing" OR "Microsoft* Copilot" OR "LLaMA" OR openai OR "Open AI" OR pathai OR "Path AI") AND ("MCQ*" OR "multiple choice question*")
